# Recent functional decline and outpatient follow-up after hospital discharge: a cohort study

**DOI:** 10.1186/s12877-023-04192-7

**Published:** 2023-09-11

**Authors:** Orly Bogler, David Kirkwood, Peter C. Austin, Aaron Jones, Chi-Ling Joanna Sinn, Karen Okrainec, Andrew Costa, Lauren Lapointe-Shaw

**Affiliations:** 1 Faculty of Medicine, University of Toronto, Toronto, Canada; 2 Institute for Clinical Evaluative Sciences McMaster, Hamilton, Canada; 3 Institute for Clinical Evaluative Sciences, Toronto, Canada; 4 Institute of Health Policy, Management and Evaluation, University of Toronto, Toronto, ON Canada; 5 Department of Health Research Methods, Evidence, and Impact, McMaster University, Hamilton, Canada; 6 Toronto General Hospital Research Institute, Department of Medicine, University Health Network, Toronto, ON Canada

**Keywords:** Functional decline, Hospital discharge, Physician follow-up, Health services, Homecare, Readmissions

## Abstract

**Background:**

Functional decline is common following acute hospitalization and is associated with hospital readmission, institutionalization, and mortality. People with functional decline may have difficulty accessing post-discharge medical care, even though early physician follow-up has the potential to prevent poor outcomes and is integral to high-quality transitional care. We sought to determine whether recent functional decline was associated with lower rates of post-discharge physician follow-up, and whether this association changed during the COVID-19 pandemic, given that both functional decline and COVID-19 may affect access to post-discharge care.

**Method:**

We conducted a retrospective cohort study using health administrative data from Ontario, Canada. We included patients over 65 who were discharged from an acute care facility during March 1st, 2019 – January 31st, 2020 (pre-COVID-19 period), and March 1st, 2020 – January 31st, 2021 (COVID-19 period), and who were assessed for home care while in hospital. Patients with and without functional decline were compared. Our primary outcome was any physician follow-up visit within 7 days of discharge. We used propensity score weighting to compare outcomes between those with and without functional decline.

**Results:**

Our study included 21,771 (pre-COVID) and 17,248 (COVID) hospitalized patients, of whom 15,637 (71.8%) and 12,965 (75.2%) had recent functional decline. Pre-COVID, there was no difference in physician follow-up within 7 days of discharge (Functional decline 45.0% vs. No functional decline 44.0%; RR = 1.02, 95% CI 0.98–1.06). These results did not change in the COVID-19 period (Functional decline 51.1% vs. No functional decline 49.4%; RR = 1.03, 95% CI 0.99–1.08, Z-test for interaction p = 0.72). In the COVID-19 cohort, functional decline was associated with having a 7-day physician virtual visit (RR 1.15; 95% CI 1.08–1.24) and a 7-day physician home visit (RR 1.64; 95% CI 1.10–2.43).

**Conclusions:**

Functional decline was not associated with reduced 7-day post-discharge physician follow-up in either the pre-COVID-19 or COVID-19 periods. In the COVID-19 period, functional decline was positively associated with 7-day virtual and home-visit follow-up.

**Supplementary Information:**

The online version contains supplementary material available at 10.1186/s12877-023-04192-7.

## Introduction

In the time surrounding hospitalization for an acute medical illness, older adults (≥ 65 years of age) often experience functional decline in both their basic and instrumental activities of daily living [1–6], and are at increased risk of becoming homebound [7–9]. Hospital-associated functional decline is often multifactorial and can be attributed to malnutrition, immobilization, acute medical symptoms, cognitive impairment, and pressure ulcers [10, 11]. Functional decline and being homebound have been associated with an increased risk of hospital readmission [12], institutionalization [13], and death [14–17].

Transitional care programs were developed to reduce the risk of hospital readmission [18, 19]. Early outpatient physician follow-up is a core component of many transitional care bundles [19–23]. The evidence supporting the benefit of early physician follow-up for preventing readmissions is strongest for patients with chronic obstructive pulmonary disease (COPD) and congestive heart failure (CHF), where rates of readmission post-hospitalization are high [20, 24–30]. Post-discharge follow-up programs have also been successful at reducing hospital readmissions in other patient populations, including those recovering from stroke, acute coronary syndrome, and surgical procedures [19, 31–33]. Given these benefits, timely primary care follow-up after discharge is tracked as an indicator of health care quality [34].

Post-discharge follow-up rates in older patients are quite variable, ranging between 25% and 57% [29, 35–37]. Though several studies have investigated factors that contribute to low rates of primary care follow-up after discharge [35, 36], no study has examined whether functional decline impacts this outcome. Functional impairment can present additional barriers to getting to an appointment, as a result of decreased mobility, increased reliance on others, and transportation challenges [38]. With a recent functional decline, patients and caregivers may not have had time to establish compensatory processes and support systems to account for newly acquired mobility deficits. Our objective was to investigate the relationship between recent functional decline and timely family physician follow-up after hospital discharge. Furthermore, given the notable shift to virtual care during the COVID-19 pandemic [39–41], we examined whether this relationship changed in the year following the onset of COVID-19.

## Methods

### Setting and study design

This was a retrospective cohort study using population-based administrative data housed at ICES for all residents of Ontario, Canada with a valid health insurance card. ICES is an independent, non-profit research institute whose legal status under Ontario’s health information privacy law allows it to collect and analyze health care and demographic data, without consent, for health system evaluation and improvement. Ontario has universal health insurance coverage (without premium or co-pay) for hospital and essential physician services, and as such, ICES studies are considered population-based. Patient-level data were de-identified and linked across datasets using unique encoded patient identifiers. ICES databases used in this study are listed in Appendix Table 1. Case definitions are also provided in Appendix Table 2. The use of the data in this project is authorized under Sect. 45 of Ontario’s Personal Health Information Protection Act (PHIPA) and does not require review by a Research Ethics Board.

### Population

We included patients over 65 who were discharged from an acute care hospital and who received an interRAI Contact Assessment (interRAI CA) while in hospital. The interRAI CA is a brief, standardized multidimensional tool that is part of the internationally-validated suite of interRAI instruments [42]. In Ontario, the interRAI CA is used to assess patients’ home care needs prior to discharge from hospital [43]. In Ontario, publicly funded homecare services include a mix of personal support (e.g., help with activities of daily living), and non-physician professional services (e.g., nursing, physiotherapy). An assessment for home care (and completion of an interRAI CA) occurs after a request by any member of the clinical care team (e.g. physician, nurse, physiotherapist, occupational therapist, social worker, or other).

**Fig. 1:**
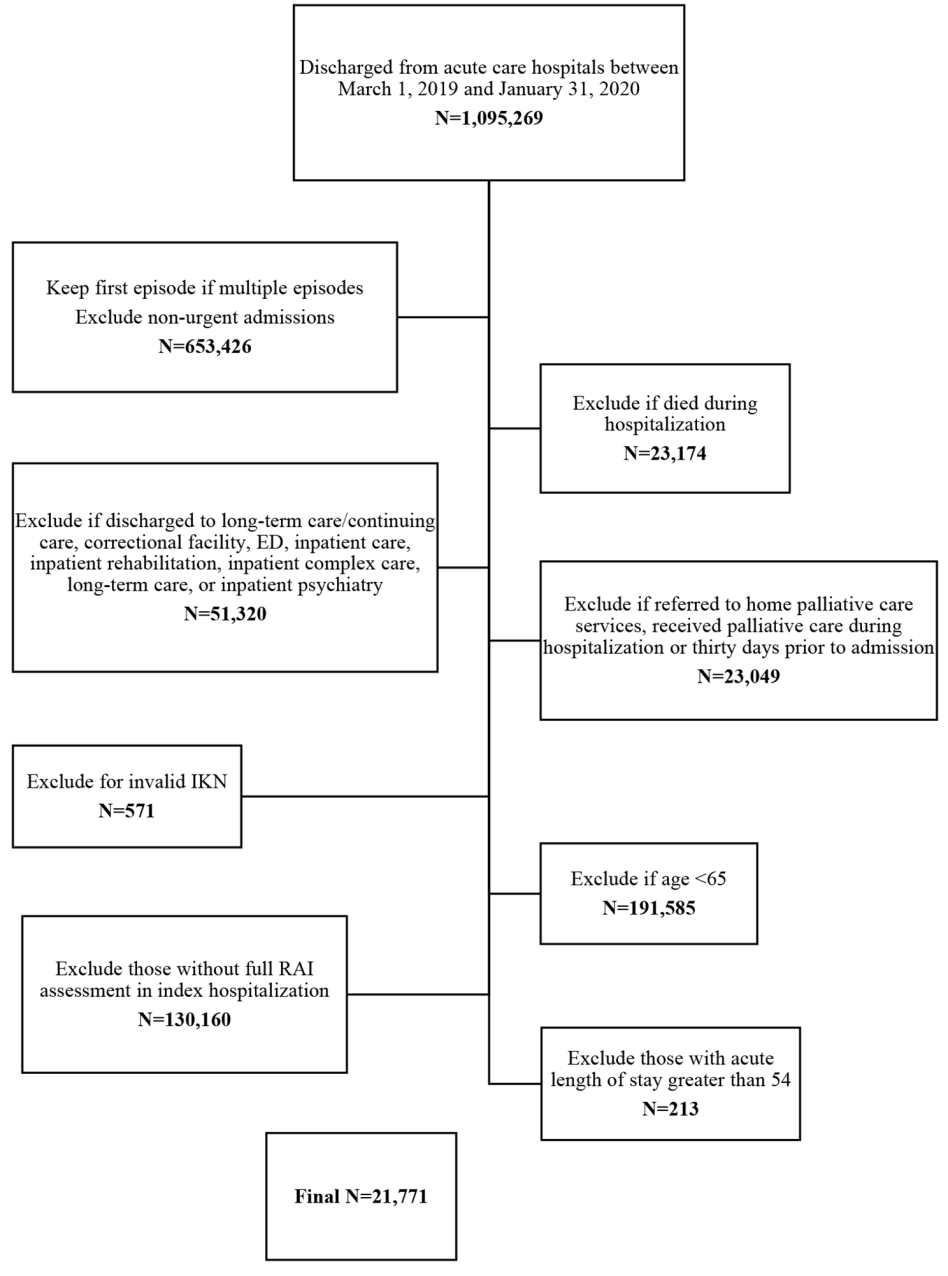
Pre-COVID-19 Cohort inclusion/exclusion criteria

**Fig. 2:**
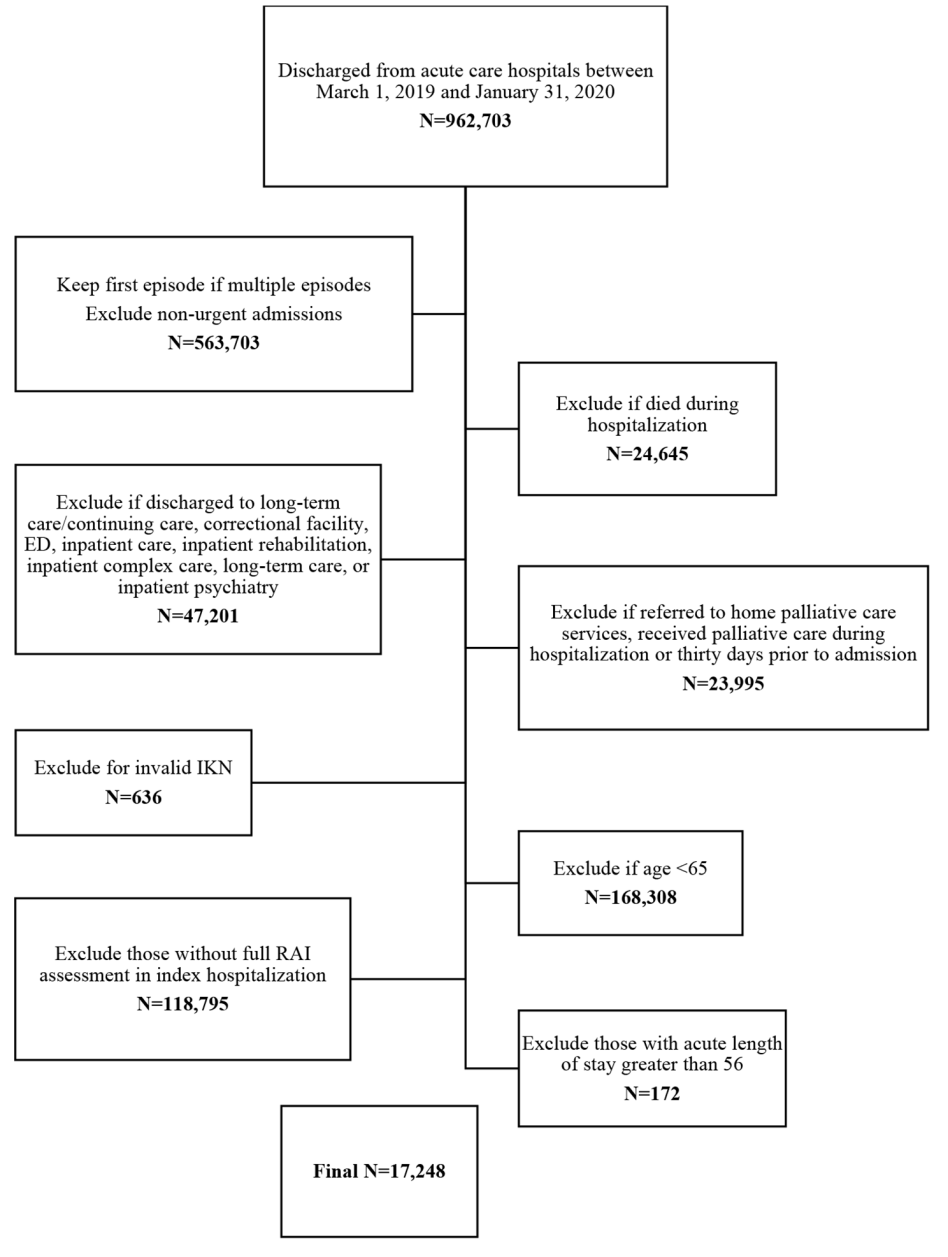
COVID-19 Cohort inclusion/exclusion criteria

We created two separate cohorts: the first included patients discharged between March 1st, 2019 and January 31st, 2020 (pre-COVID-19 cohort) and the second included patients discharged between March 1st, 2020 and January 31st, 2021 (COVID-19 cohort). We excluded elective hospitalizations, patients who died during the hospitalization, and groups that would be less likely to attend a physician office visit: those who were discharged to any place other than the community, and those that were referred to home care for end-of-life services or received palliative care within 30 days of discharge (see Figs. 1 and 2). We excluded any participants with missing or invalid age, sex, and Ontario postal code, as well as those with an acute length of stay (LOS) > 99th percentile (54 days in pre-COVID-19 cohort, 56 days in COVID-19 cohort) to achieve better balance between exposure groups for the analysis.

### Exposure

The primary exposure was recent change in functional status (“recent functional decline”), as recorded on the in-hospital interRAI CA. The specific item used for this variable was D5: “*Change in ADL status as compared to 90 days ago, or since last assessment if less than 90 days ago”* with response options of *“improved, no change, uncertain, or declined”*). We excluded any participants who were not assessed in hospital or did not complete the full interRAI CA.

The full interRAI CA contains eight ADL/IADL questions and one ADL change item. Individually, the ADL items have high interrater reliability values, and the ADL scales have been validated against the Functional Independence Measure (FIM) and Barthel Index [44, 45]. The single item “change in ADL status” captures change in ADLs only, which is primarily used in home care to identify those who may benefit from rehabilitation [46]. Change is defined as either a change in the person’s capacity for involvement in self-care or actual involvement in self-care and is based on triangulation of the person and family’s self-report and assessor observation and judgment. The interRAI CA manual instructs operators to “ask the person to think about how well he or she was able to perform ADLs 90 days ago. For example, if the person visited a family member 3 months ago, ask how capable he or she was of eating, walking, etc., during that visit. This item includes all activities of daily living (that is, bed mobility, transfer, locomotion, dressing, personal hygiene, toilet use, eating, and bathing)”. We elected to focus specifically on a *change* in baseline functional status, given that community-dwelling patients already living with baseline functional impairment may have found ways to have their care needs met, including accessing post-discharge medical care. Yet, those with recent functional decline may not yet have developed adaptive processes and support systems that would allow them to access healthcare.

We compared those with a response of “declined” (and this group is referred to throughout as patients with functional decline) to those with all other responses including improved, no change, or uncertain (this group is referred to throughout the manuscript as patients with no functional decline). The “no functional decline” was expected to include many individuals with stable functional impairment. Of note, we could not determine from our datasets whether functional decline pre-dated the index admission illness or not.

### Patient characteristics

We examined the following patient characteristics: age, sex, rural residence status [47], neighbourhood income quintile, arrival by ambulance, Charlson comorbidity score [48], acute hospital length of stay, weekend discharge, a history of CHF [49], COPD [50], or dementia [51], most responsible diagnosis (for the 20 most common diagnoses), discharge home with support services, and previous healthcare usage (emergency department visits and hospital discharges within the last six months, and personal support home care usage in the last six months). We also included several variables from the interRAI CA, including expected living arrangement, primary language, need for an interpreter, performance in activities of daily living (basic and instrumental), ability to understand others, falls in the last 90 days, absence of informal caregiver/helper, and instability of medical conditions, as measured by the Changes in Health, End-stage disease, Signs, and Symptoms scale [52].

### Outcomes

The primary outcome was the occurrence of an outpatient visit with any physician conducted virtually, at home, or in office, within 7 days of discharge (Appendix Table 2). This discrete time period was chosen to align with the 7-day physician follow-up quality indicator used by Health Quality Ontario [34]. Secondary outcomes included virtual, home, or office physician follow-up at 7-, 14- and 30-days post-discharge (Appendix Table 2) and time-to-home care visit. We also included death, urgent readmissions, transfer to a long-term care facility, and emergency department visits within 30 days of discharge, as well as whether an emergency department visit occurred after discharge but prior to first physician follow-up visit.

### Analysis

We used propensity score weighting methods to minimize the effects of confounding in our analysis [53]. The propensity score was estimated using a logistic regression model in which functional decline was regressed on age, sex, rural status, neighbourhood income quintile, arrival by ambulance, Charlson comorbidity score, a history of dementia, CHF, or COPD, acute length of stay, previous home care usage (6 months), previous emergency department visits (6 months), previous hospital discharge (6 months), top 20 most responsible diagnoses, whether an interpreter was needed during the assessment, whether they lived alone, and the absence of an informal helper.

For this analysis, we estimated the average treatment effect among the treated population (ATT) using propensity score ATT weights. We assigned a weight equal to 1 to those with functional decline whereas patients without functional decline were assigned a weight equal to the odds of exposure (*PS/(1-PS)*), where PS denotes the estimated propensity score [53]. We compared the balance of baseline covariates between groups in the weighted sample using standardized mean differences [54].

To estimate the association of functional decline with binary outcomes, we calculated relative risks (RRs) directly in the weighted sample and obtained confidence intervals using 2,000 bootstrap samples. We used bootstrap estimates of standard errors, given that these estimates work as well as or better than the asymptotic standard errors when using PS weighting [55]. To estimate whether there were significant differences in the association of functional decline with outcomes between the pre-COVID-19 and COVID-19 time periods, we used a Z-test to compare the difference of the log of each RR between the two time periods (with the standard error of the log-relative risk computed using standard deviation of the log-relative risk across the 2,000 bootstrap samples). For time-to-home care, we calculated hazard ratios using a weighted Cox regression model with robust standard errors to understand the association of functional decline with time to home care after hospital discharge, censored at 30 days. In response to reviewer feedback, we conducted a post-hoc analysis in which we compared the unweighted counts of individuals with and without 7-day virtual physician follow-up in the pre-COVID and COVID periods, among those with functional decline, using a Chi-square test.

Significance was defined as p < 0.05 and all hypothesis testing was two-tailed. All analyses were performed in SAS software, version 9.4 (SAS Institute Inc.).

## Results

### Pre-COVID-19 cohort

#### Baseline characteristics

Of the 21,771 patients in the pre-COVID-19 cohort, 15,637 (71.8%) had recent functional decline. Prior to weighting, those with and without functional decline had standardized differences that exceeded 10% for age, comorbidity count, dementia, ADL impairment, unstable health conditions, and recent falls (Appendix Table 3). The greatest difference between groups with regards to ADL impairment was ability to bathe independently; 84.1% of those with functional decline required supervision compared to only 48% in those without functional decline (SMD = 0.83). After weighting, the greatest differences between groups were in the acute length of stay (SMD = 0.07) and presence of dementia (SMD = 0.02), which were, respectively, longer, and more prevalent among those without recent functional decline (Table 1). The weighted standardized differences between the characteristics of the two groups were all less than 10% (Table 1).

**Table 1:** Characteristics of the Propensity-Score Weighted Pre-COVID-19 Cohort: Patients with Functional Decline and Controls

Variable	Functional Decline (N = 15,637)	Controls (N = 15,810)	Weighted SMD^1^
Age (Mean)	81.5	81.4	0.01
Female (n, %)	9,217 (58.9)	9,438.22 (59.7)	0.02
Rural Status (n, %)			
- Large urban - Small urban - Rural - Missing	10,038 (64.2) 4,164 (26.6) 1,293 (8.3) 142 (0.9)	10,132.4 (64.1) 4,217.5 (26.7) 1,307.6 (8.3) 152.8 (0.9)	0.00 0.00 0.00 0.01
Income Quintile (n, %)			
- 1 (Lowest) - 2 - 3 - 4 - 5 (Highest) - Missing	4,140 (26.5) 3,709 (23.7) 2,955 (18.9) 2,506 (16.0) 2,277 (14.6) 50 (0.3)	4,206.1 (26.6) 3,838.5 (24.3) 2,924.4 (18.5) 2,450.3 (15.5) 2,336.5 (14.8) 54.6 (0.3)	0.00 0.01 0.01 0.01 0.01 0.00
Arrived by ambulance (n, %)	10,503 (67.2)	10,713.9 (67.8)	0.01
Charlson Index Group (n, %)			
- 0 - 1 - 2 - 3 - 4+	5,347 (34.2) 4,269 (27.3) 2,918 (18.7) 1,910 (12.2) 1,193 (7.6)	5,545.0 (35.1) 4,333.9 (27.4) 2,835.9 (17.9) 1,889.2 (12.0) 1,206.4 (7.6)	0.02 0.00 0.02 0.01 0.00
Dementia (n, %)	4,338 (27.7)	4,508.6 (28.5)	0.02
CHF (n, %)	4,213 (26.9)	4,234.6 (26.8)	0.00
COPD (n, %)	6,092 (39.0)	6,158.0 (39.0)	0.00
Acute Length of Stay (Mean)	10.18	10.8	0.07
Homecare usage in 6 months prior to admission (n, %)	5,842 (37.4)	5,950.1 (37.6)	0.01
Previous Emergency Department visit in 6 months prior to admission (n, %)	9,521 (60.9)	9,692.7 (61.3)	0.01
Hospital discharge in 6 months prior to admission (n, %)	1,471 (9.4)	1,545.1 (9.8)	0.01
Interpreter needed (n, %)	1,505 (9.6)	1,526.5 (9.7)	0.00
Living alone (n, %)	4,865 (31.1)	5,022.8 (31.8)	0.01
Absent informal helper (n, %)	339 (2.2)	345.1 (2.2)	0.00
Top 20 most responsible diagnoses (n, %)			
- Congestive heart failure - Urinary tract infection - COPD exacerbation - COPD with acute lower respiratory infection - Pneumonia - Acute renal failure - Delirium - NSTEMI - Femoral neck fracture - Cellulitis - Dementia - Cerebral infarction, unspecified - Cerebral infarction due to occlusion/stenosis of cerebral artery - Delirium superimposed on dementia - Intertrochanteric fracture - Atrial fibrillation - Convalescence following surgery - Malaise and fatigue - Falls - Sepsis	1,028 (6.6) 512 (3.3) 391 (2.5) 354 (2.3) 414 (2.7) 274 (1.8) 269 (1.7) 244 (1.6) 294 (1.9) 194 (1.2) 246 (1.6) 196 (1.3) 210 (1.3) 212 (1.4) 216 (1.4) 148 (1.0) 137 (0.9) 170 (1.1) 167 (1.1) 152 (1.0)	1,001.4 (6.3) 521.4 (3.3) 391.3 (2.5) 333.5 (2.1) 423.9 (2.7) 266.9 (1.7) 274.6 (1.7) 243.5 (1.5) 312.0 (2.0) 185.8 (1.2) 291.0 (1.8) 178.4 (1.1) 198.3 (1.3) 224.80 (1.4) 248.0 (1.6) 161.3 (1.0) 134.7 (0.9) 177.3 (1.1) 181.0(1.1) 161.2 (1.0)	0.01 0.00 0.00 0.01 0.00 0.01 0.00 0.00 0.01 0.01 0.02 0.01 0.01 0.01 0.02 0.01 0.00 0.00 0.01 0.01

^1^ SMD = Standardized Mean difference

#### Outcomes

In the propensity score-weighted sample, there was no difference in the primary outcome of any physician follow-up within 7 days of discharge (Functional decline 45.0% vs. No functional decline 44.0%; RR = 1.02, 95% CI 0.98–1.06). Patients with functional decline were more likely to have a physician follow-up visit within 14 days of discharge (71.3% vs. 69.5%; RR = 1.03, 95% CI 1.00–1.05), but there was no difference at 30 days (87.8% vs. 87.5%; RR = 1.00, 95% CI 0.99–1.02). Patients with functional decline were also more likely to have a follow-up visit at their home (3.5% vs. 2.5%; RR = 1.37, 95% CI 1.09–1.72). There were no differences in the proportion with 7, 14, or 30-day post-discharge specialist, family physician, or previously known family physician follow-up (Table 2).

Patients with functional decline were also more likely to be transferred to long-term care facility (2.1% vs. 1.3%; RR = 1.61, 95% CI 1.15–2.26) and die within 30 days of discharge (2.9% vs. 1.9%; RR = 1.55, 95% CI 1.22–1.98). There were no differences between the groups for 30-day hospital readmission, emergency department visit, emergency department visit prior to first follow-up visit, and time to home care (Table 2).

**Table 2:** Outcomes in Patients with Functional Decline compared to Propensity-Score Weighted Controls, in Pre-COVID-19 and COVID-19 Cohorts

	Pre-COVID-19 Cohort			COVID-19 Cohort			P value for Z-test for interaction^3^
Outcome	Functional Decline (N = 15,637)	Control (N = 15,810)	RR^1^ (95% CI)	Functional Decline (N = 12,965)	Control (N = 13,132)	RR^2^ (95% CI)	
Physician follow-up visit (n, %)							
- Within 7 days of discharge - Within 14 days of discharge - Within 30 days of discharge	7042 (45.0) 11,154 (71.3) 13,721 (87.8)	6962.3 (44.0) 10986.9 (69.5) 13834.7 (87.5)	1.02 (0.98–1.06) 1.03 (1.00-1.05)^*^ 1.003 (0.99–1.02)	6619 (51.1) 9452 (72.9) 11,354 (87.6)	6486.1 (49.4) 9542.1 (72.7) 11498.7 (87.6)	1.03 (0.99–1.08) 1.00 (0.98–1.03) 1.00 (0.99–1.02)	0.72 0.20 0.80
Physician follow-up visit within 7 days of discharge, within each of the following settings (n, %)							
- Office - Virtual - Home	3869 (24.7) 34 (0.2) 544 (3.5)	3972.6 (25.1) 28.1 (0.2) 401.9 (2.5)	0.99 (0.93–1.04) 1.22 (0.59–2.55) 1.37 (1.09–1.71)^*^	1301 (10.03) 3803 (29.3) 245 (1.9)	1436.5 (10.9) 3338.1 (25.4) 151.7 (1.2)	0.92 (0.82–1.03) 1.15 (1.08–1.24)^*^ 1.64 (1.10–2.43)^*^	0.28 0.88 0.44
Family physician follow-up visit (n, %)							
- Within 7 days of discharge - Within 14 days of discharge - Within 30 days of discharge	4298 (27.5) 7679 (49.1) 10,527 (67.3)	4178.2 (26.4) 7711.6 (48.8) 10658.5 (67.4)	1.04 (0.98–1.10) 1.01 (0.97–1.04) 1.00 (0.98–1.02)	4537 (35.0) 6912 (53.3) 8909 (68.7)	4205.9 (32.0) 6870.4 (52.3) 8827.6 (67.2)	1.09 (1.02–1.17)^*^ 1.02 (0.98–1.06) 1.02 (0.99–1.06)	0.27 0.67 0.26
Specialist follow-up visit (n, %)							
- Within 7 days of discharge - Within 14 days of discharge - Within 30 days of discharge	2744 (17.6) 4218 (27.0) 5150 (32.9)	2784.1 (17.6) 4088.1 (25.9) 5158.7 (32.6)	1.00 (0.93–1.07) 1.04 (0.98–1.11) 1.01 (0.96–1.06)	2082 (16.1) 3096 (23.9) 3818 (29.5)	2280.2 (17.4) 3312.2 (25.2) 4129.6 (31.5)	0.93 (0.84–1.02) 0.95 (0.88–1.03) 0.94 (0.88-1.00)	0.23 0.05 0.08
Follow-up visit with known family physician (n, %)							
- Within 7 days of discharge - Within 14 days of discharge - Within 30 days of discharge	3165 (20.2) 5198 (33.2) 6304 (40.3)	3162.2 (20.0) 5334 0.2 (33.7) 6458.1 (41.0)	1.01 (0.95–1.08) 0.99 (0.94–1.03) 0.98 (0.94–1.02)	3544 (27.3) 4960 (38.3) 5793 (44.7)	3343.0 (25.5) 4952.9 (37.7) 5711.7 (43.5)	1.07 (0.99–1.17) 1.01 (0.96–1.08) 1.03 (0.98–1.08)	0.28 0.45 0.19
Urgent readmission to hospital within 30 days of discharge (n, %)	2552 (16.3)	2449.5 (15.5)	1.05 (0.97–1.14)	2061 (15.9)	1883.6 (14.3)	1.11 (0.99–1.23)	0.46
Death within 30 days of discharge (n, %)	459 (2.9)	298.8 (1.9)	1.55 (1.22–1.98)^*^	406 (3.1)	360.4 (2.7)	1.14 (0.85–1.53)	0.11
Emergency Department visit within 30 days of discharge (n, %)	4249 (27.2)	4292.1 (27.2)	1.00 (0.95–1.06)	3262 (25.2)	3342.8 (25.5)	0.99 (0.92–1.06)	0.78
Emergency Department visit after discharge but before first follow-up physician visit (n, %)	642 (4.1)	685.4 (4.3)	0.95 (0.81–1.10)	369 (2.9)	565.0 (4.3)	0.66 (0.51–0.86)^*^	0.02^*^
Transferred to long-term care facility within 30 days of discharge (n, %)	323 (2.1)	202.4 (1.3)	1.61 (1.15–2.26)^*^	62 (0.5)	55.8 (0.4)	1.13 (0.31–4.05)	0.59
	**Functional decline**	**Control**	**HR** ^**1**^ **(95% CI)**	**Functional Decline**	**Control**	**HR** ^**2**^ **(95% CI)**	**P-value for interaction test**
Time to homecare visit post-discharge (mean, 95% CI)	0.61 (0.57–0.64)	0.66 (0.59–0.72)	1.02 (1.00-1.04)	0.59 (0.56–0.63)	0.66 (0.57–0.75)	1.02 (0.99–1.05)	0.92

^1^Outcomes of propensity-weighted matched cohort in pre-COVID time-period (comparing functional decline to no functional decline)

^2^Outcomes of propensity-weighted matched cohort in COVID time-period (comparing functional decline to no functional decline)

^3^P-value for Z-test comparing RRs of each time-period (test of interaction – COVID vs. Pre-COVID).

*Denotes significance

### COVID-19 cohort

#### Baseline characteristics

There were 17,248 patients in the COVID-19 cohort who were discharged from hospital between March 1, 2020, and January 31, 2021 (Fig. 2). Of these patients, 12,965 (75.2%) had functional decline. Prior to weighting, patients with functional decline were older (81.2 vs. 79.3 years) and more likely to have dementia and to be dependent for all ADLs (Appendix Table 4). Consistent with the pre-COVID cohort, the greatest difference between groups with regards to ADL impairment was ability to bathe independently (SMD = 0.88). After weighting, the variables with the highest standardized differences included acute length of stay (SMD = 0.09) and presence of dementia (SMD = 0.03) (Table 3).

**Table 3:** Characteristics of the Propensity Score Weighted COVID-19 Cohort: Patients with Functional Decline and Controls

Variable	Functional Decline (N = 12,965)	Control (N = 13,132)	Weighted SMD^1^
Age (Mean)	81.18	81.0	0.02
Female (n, %)	7,505 (57.9)	7,732.9 (58.9)	0.02
Rural Status (n, %)			
- Large urban - Small urban - Rural - Missing	8,174 (63.0) 3,499 (27.0) 1,147 (8.9) 145 (1.1)	8,191.4 (62.4) 3,560.5 (27.1) 1,235.9 (9.4) 144.6 (1.1)	0.01 0.00 0.02 0.00
Income Quintile (%)			
- 1 (Lowest) - 2 - 3 - 4 - 5 (Highest) - Missing	3,332 (25.7) 2,955 (22.8) 2,492 (19.2) 2,146 (16.5) 2,004 (15.5) 36 (0.3)	3456.7 (26.3) 3019.4 (23.0) 2387.0 (18.2) 2182.5 (16.6) 2054.3 (15.6) 32.5 (0.3)	0.01 0.01 0.03 0.00 0.01 0.01
Arrived by ambulance (%)	9,330 (72.0)	9,578.2 (73.0)	0.02
Charlson Index Group ( %)			
- 0 - 1 - 2 - 3 - 4+	4,541 (35.0) 3,462 (26.7) 2,460 (19.0) 1,571 (12.1) 931 (7.2)	4,647.7 (35.4) 3,455.1 (26.3) 2,454.1 (18.7) 1,543.8 (11.8) 1,031.8 (7.9)	0.01 0.01 0.01 0.01 0.03
Dementia (n, %)	2,316 (17.9)	2,499.4 (19.0)	0.03
CHF (n, %)	2,674 (20.6)	2,735.4 (20.8)	0.01
COPD (n, %)	4,341 (33.5)	4,522.7 (34.4)	0.02
Acute Length of Stay (Mean)	10.9	11.8	0.09
Homecare usage in 6 months prior to admission (n, %)	4,648 (35.9)	4,826.6 (36.8)	0.02
Previous emergency department visit in 6 months prior to admission (n, %)	7,390 (57.0)	7,486.9 (57.0)	0.00
Hospital discharge in 6 months prior to admission (n, %)	1,142 (8.8)	1,163.3 (8.9)	0.00
Interpreter needed (n, %)	1,208 (9.3)	1,143.3 (8.7)	0.02
Living alone (n, %)	3,965 (30.6)	4,141.6 (31.5)	0.02
Absent informal helper (%)	294 (2.3)	299.8 (2.3)	0.00
Top 20 most responsible diagnoses (%)			
- Congestive heart failure - Urinary tract infection - Delirium - Femoral neck fracture - Acute renal failure - COVID-19 - COPD exacerbation - Intertrochanteric fracture - COPD with acute lower respiratory infection - Pneumonia - NSTEMI - Delirium superimposed on dementia - Cerebral infarction due to occlusion/stenosis of cerebral artery - Cerebral infarction, unspecified - Dementia - Cellulitis - Convalescence following surgery - Atrial fibrillation - Malaise and fatigue - Sepsis	669 (5.2) 466 (3.6) 287 (2.2) 309 (2.4) 244 (1.9) 274 (2.1) 196 (1.5) 296 (2.3) 186 (1.4) 200 (1.5) 180 (1.4) 215 (1.7) 204 (1.6) 182 (1.4) 190 (1.5) 143 (1.1) 133 (1.0) 129 (1.0) 137 (1.1) 116 (0.9)	653.5 (5.0) 456.3 (3.5) 286.4 (2.2) 333.3 (2.5) 252.3 (1.9) 310.2 (2.4) 202.3 (1.5) 381.7 (2.9) 196.5 (1.5) 208.2 (1.6) 181.1 (1.4) 208.2 (1.6) 188.5 (1.4) 188.6 (1.4) 260.8 (2.0) 148.3 (1.1) 131.5 (1.0) 136.3 (1.0) 136.6 (1.0) 110.4 (0.8)	0.01 0.01 0.00 0.01 0.00 0.02 0.00 0.04 0.01 0.00 0.00 0.01 0.01 0.00 0.04 0.00 0.00 0.00 0.00 0.01

^1^ SMD = Standardized Mean difference

#### Outcomes

In the propensity weighted sample, there was no difference in the primary outcome of physician follow-up within seven days of discharge (Functional decline 51.1% vs. No functional decline 49.4%; RR = 1.03, 95% CI 0.99–1.08). There were also no differences in follow-up rates within 14 and 30 days of discharge. Patients with functional decline were more likely to have a virtual visit compared to those without functional decline (29.3% vs. 25.4%; RR = 1.15, 95% CI 1.08–1.24), and more likely to have a visit at home (1.9% vs. 1.2%; RR = 1.64, 95% CI 1.10–2.43). Patients with functional decline were more likely to have a family physician visit within 7 days of discharge (35.0% vs. 32.0%; RR = 1.09, 95% CI 1.02–1.17). Otherwise, like the pre-COVID-19 cohort, there were no differences in specialist, family physician, or previously known family physician visits at 7, 14, or 30-days post-discharge time periods for specialist, (Table 3).

Similar to the pre-COVID-19 cohort, there were no differences found between the time to home care initiation between those with functional decline and those without. In contrast to the pre-COVID-19 cohort, there were no differences between groups in mortality or transfer to a long-term care facility within 30 days post-discharge. Patients with functional decline were less likely to have an emergency department visit that occurred prior to their first follow-up visit (RR = 0.66, 95% CI 0.51–0.86) (Table 3).

### Effect modification by timing relative to COVID-19

The association between functional decline and the primary outcome was not modified by the timing relative to COVID-19 (Z=-0.36, p = 0.72). Similarly, there was no difference in any follow-up outcomes at 7-, 14- or 30-days post-discharge. Patients in the COVID-19 period were less likely to have an emergency department visit prior to their first follow-up visit (Z = 2.33, p = 0.02).

In contrast to the pre-COVID period, where only 0.2% (N = 34/15,637) of those with functional decline had a virtual physician follow-up within 7 days, in the COVID period this was 29.3% (N = 3803/12,965, p = 0.0001).

## Discussion

Among hospitalized patients over 65 who were assessed for home care services, recent functional decline was not associated with lower rates of physician follow-up within 7 days of hospital discharge. In the pre-COVID-19 cohort, functional decline was associated with a greater likelihood of a follow-up visit at home, and death or transfer to long-term care facility within 30 days of discharge. In the COVID-19 cohort, functional decline was associated with a greater likelihood of having a virtual or home-based follow-up visit, and with having a family physician visit within 7 days of discharge.

To our knowledge, this is the first study to examine the association between functional decline and post-discharge physician follow-up. Many patients experience functional decline leading up to and during hospitalization, and this is associated with greater reliance on supports in the community, higher rates of institutionalization, hospital readmission, and mortality [56–59]. Our finding of no association between functional decline and follow-up is good news; it means that a group that could face challenges with accessing post-discharge care is adequately supported during the transition home. Because everyone in our cohort had a RAI-CA home care assessment, both exposure groups had high levels of comorbidity, previous home care use, and functional impairment. Providers may be equally likely to arrange timely follow-up among populations perceived to be more vulnerable or at-risk, especially at transitional periods, regardless of whether or not there was a recent functional decline. This is supported by our study’s follow-up attendance rates of almost 50% at 7-days post-discharge and 90% at 30-days post-discharge – rates that are higher than for the general population, which range from 25 to 57% [29, 35–37]. Although overall 7-day follow-up rates were not different between the two groups, patients with functional decline received more virtual and home visits, suggesting that they were prioritized for more accessible appointment types.

Our findings that patients with functional decline had a higher risk of death within 30 days of discharge and transfer to long-term care facility within 30 days of discharge are consistent with previous literature [13–15]. Interestingly, this association was not present in the COVID-19 cohort; a possible explanation for this is that during the COVID-19 pandemic patients and families avoided long-term care facilities due to frequent COVID-19 outbreaks in these settings [60]. Notably, patients in the COVID-19 cohort were also less likely to have an emergency department visit that occurred prior to their first follow-up (RR = 0.66; 95% CI 0.51–0.86). This is possibly reflective of emergency department avoidance during COVID-19 [61], particularly among those with recent functional decline.

One of our study aims was to determine whether the COVID-19 pandemic, and the resulting widespread availability of virtual care, impacted follow-up appointment attendance among patients with functional decline. Though we did not set out to test whether the proportion of patients who had their follow-up delivered virtually changed from pre-COVID-19 to during COVID, we observed that the rate of 7-day virtual follow-up increased 100-fold between the time periods. Our findings are consistent with a recent study in a US Medicare population that showed similar 30-day outpatient post-discharge follow-up rates from April 2019 to April 2020, with an increase in virtual visits between time periods [62]. This highlights that virtual post-discharge follow-up has been integrated into transitional care processes for older adults. This is fortunate, as virtual visits may have been the only way for this population to access post-discharge care during the pandemic.

Many patients may prefer an in-person post-discharge visit with their primary care provider rather than a phone call [63]. Our study did not examine the relationship between the use of telemedicine and patient outcomes, and we cannot conclude whether the substitution of in-person visits by virtual visits had a positive, neutral, or negative impact on hospital readmissions. In a recent study of Medicare beneficiaries, in-person follow-up was associated with fewer 30-day readmissions than virtual follow-up [64]. In another study of patients with heart failure, there was no such difference [65]. Further research is needed to determine the impact of timing and type of follow-up attendance on post-discharge outcomes, including readmission rates and patient experience.

Our study has several strengths and limitations. A primary strength of the study was our methodology and analysis; we used a large administrative dataset and propensity-score weighting, which allowed us to control for and minimize confounding due to measured characteristics. However, we could not identify appointments that were scheduled but not attended, as administrative data sources only capture billed (i.e., completed) visits. We also could not determine the exact timing or magnitude of functional decline, including whether it pre-dated their illness and/or hospitalization. Additionally, we excluded patients whose length of stay exceeded the 99th percentile, who also may have been more likely to have functional decline- as this was less than 1% of our sample, this exclusion would not be expected to alter our overall results. The wide confidence intervals for several of our secondary outcomes (7-day virtual or home follow-up, long-term care facility admission) suggest that there was inadequate power to detect small differences in these outcomes, or to test for interaction in the pre- and post-COVID-19 periods. All analyses of secondary outcomes were exploratory in nature. Lastly, to ensure internal validity, our study population was focused on those being assessed for home care using the interRAI CA, a population with high levels of baseline functional impairment. Our finding of a lack of association between recent functional decline and follow-up attendance may not be generalizable to healthier inpatient populations.

## Conclusion

Overall, our study found that among patients over age 65 being assessed in hospital for home care, recent functional decline did not impair access to post-discharge follow-up visits. COVID-19 was accompanied by a substantial increase in virtual visits, which likely played an important role in preserving access to post-discharge medical care for adults with recent functional decline. Further research is needed to determine the impact of timing and type of follow-up attendance on post-discharge outcomes, including readmission rates and patient experience.

### Electronic supplementary material

Below is the link to the electronic supplementary material.

Supplementary Material 1

## Data Availability

The dataset from this study is held securely in coded form at ICES. While legal data sharing agreements between ICES and data providers (e.g., healthcare organizations and government) prohibit ICES from making the dataset publicly available, access may be granted to those who meet pre-specified criteria for confidential access, available at https://www.ices.on.ca/DAS (email: das@ices.on.ca). The full dataset creation plan and underlying analytic code are available from the authors upon request (via the corresponding author Orly Bogler, orly.bogler@mail.utoronto.ca), understanding that the computer programs may rely upon coding templates or macros that are unique to ICES and are therefore either inaccessible or may require modification. This document used data adapted from the Statistics Canada Postal Code^OM^ Conversion File, which is based on data licensed from Canada Post Corporation, and/or data adapted from the Ontario Ministry of Health Postal Code Conversion File, which contains data copied under license from ©Canada Post Corporation and Statistics Canada. Parts of this material are based on data and information compiled and provided by: Canadian Institute for Health Information (CIHI) and Ontario Ministry of Health (MOH). The analyses, conclusions, opinions, and statements expressed herein are solely those of the authors and do not reflect those of the funding or data sources; no endorsement is intended or should be inferred.
