# Supplementary material for: Recent functional decline and outpatient follow-up after hospital discharge: a cohort study
Source: BMC Geriatr. 2023 Sep 11;23:550. doi: 10.1186/s12877-023-04192-7 (PMC10496187; doi:10.1186/s12877-023-04192-7)
Supplement: Supplementary file 1 — Supplementary Material 1 [file 12877_2023_4192_MOESM1_ESM.docx]

APPENDIX CONTENTS

1. Table 1: ICES Dataset Details
2. Table 2: Operational Definitions for all Descriptive Variables Used
3. Table 3: Baseline Characteristics of Patients with and without Functional Decline in the Pre-COVID-19 cohort.
4. Table 4: Baseline Characteristics of Patients with and without Functional Decline in the COVID-19 Cohort.

**Table 1***.* **ICES Dataset Details**

| **Database** | **Details** |
| --- | --- |
| Registered Persons Database (RPDB) | Information about anyone with an Ontario health card number (any Ontarians alive since 1990). Main data points include demographic information and geographic information. |
| Ontario Health Insurance Plan (OHIP) | OHIP information on all billing claims submitted by Ontario physicians. |
| Discharge Abstract Database (DAD) | The DAD contains information on all admissions to acute care hospitals in Ontario including dates of admission, discharge, diagnostic codes, and procedural codes. Consecutive DAD records are linked together to form “episodes of care”. |
| Home Care Database | Information on publicly funded home care provision in Ontario including date of home care visit. |
| RAI-CA | Information on the interRAI Contact Assessment (interRAI CA), a short screening assessment completed for adults at the time of intake to home care/palliative care services, done in the community or the hospital. Its purpose is to act as a decision-aid regarding urgency of provision of services and the need for full (RAI-HC) assessment. |
| NACRS | Contains administrative, clinical (diagnoses and procedures), demographic, and administrative information for all patient visits made to hospital and  community-based ambulatory care centres (emergency departments, day  surgery units, hemodialysis units, and cancer care clinics) in Ontario. Linked to DAD to identify transitions to other care settings (i.e., inpatient acute care). |
| CENSUS | Canadian Census (2016) includes information on postal code, used for neighborhood-based information (e.g. income quintiles). |
| CHF Dataset | Contains all Ontario individuals with CHF identified since 1991. This dataset combines data from DAD, OMHRS, OHIP and NACRS. |
| COPD Dataset | Contains all Ontario COPD patients identified since 1991. This dataset combines data from DAD and OHIP. |
| Dementia Dataset | Includes all persons identified with Alzheimer’s and related dementias in ICES data holdings between ages 40 and 110 years. Combines data from DAD, OHIP, and prescribed medications from Ontario Drug Benefit (ODB). |
| Continuing Care Reporting System (CCRS) | The Continuing Care Reporting System (CCRS) contains clinical and demographic information on residents receiving facility-based continuing care services, including residents of nursing homes (long-term care facilities). |

**Table 2. Operational Definitions for all Descriptive Variables Used**

| **Variable** | **Data Source** | **Definition** |
| --- | --- | --- |
| **Descriptive Characteristics** | | |
| Age | RPDB, DAD | At time of discharge (Discharge date – Birth date) |
| Sex | RPDB | Male  Female |
| Rural status | RPDB | Postal code converted to RIO score  0-9: large urban  10-39: small urban  40+: rural |
| Income Quintile | RPDB, Census | Nearest census-based income quintile based on postal code (based on 2016 census) |
| Homeless Indicator | DAD | Yes or no |
| Arrived by ambulance | DAD | Yes or no |
| Twenty most-responsible diagnosis for index admission | DAD | Diagnostic codes. |
| Charlson Index Group | DAD | Categories: 0, 1, 2, 3, 4+ |
| Dementia |  | Presence of dementia in database |
| Congestive heart failure (CHF) | CHF | Presence of CHF in databases |
| Chronic obstructive pulmonary disease (COPD) | COPD | Presence of COPD in database |
| Length of Stay | DAD | Discharge date – Admission Date |
| Acute Length of Stay | DAD | Acute care inpatient days |
| Weekend Discharge | DAD | Discharged on a Friday, Saturday, or Sunday. |
| Homecare usage in 6 months prior to admission | HCD | Homecare encounters with personal services (PSW), homemaking services (HM), or combined PSW/HM services, that took place in a private household. |
| Previous Emergency Department visit in 6 months prior to admission | NACRS | At least one Emergency Department visit within the six months prior to admission. |
| Hospital discharge in 6 months prior to admission | DAD | Discharged from an acute care facility within the six months prior to admission. |
| Interpreter needed | RAICA | Yes or no |
| Primary language not French or English | RAICA | Yes or no |
| Living alone | RAICA | Yes or no |
| Living in a private home or apartment | RAICA | Yes or no |
| Absent informal helper | RAICA | RAICA_D19A=9 |
| Supervision or assistance needed with ADLS/iADLS | RAICA | 0=Independent  1=Supervision/assistance with task |
| Ability to understand others | RAICA | Understands (RAICA_D2FMT=0)  Usually understands (RAICA_D2FMT=1)  Often/sometimes/rarely/never understands (RAICA_D2FMT=2,3,4) |
| Instability of medical conditions | RAICA | Yes or no |
| One or more falls in last 90 days | RAICA | Yes or no |
| **Outcomes** |  |  |
| Family Physician follow-up within 7, 14, and 30 days of discharge | OHIP | Visit with OHIP specialty SPEC = 00 |
| Follow-up visit within 7/14/30 days with a previously known Family Physician | OHIP | Visit within two years prior to discharge date with the same PHYSNUM as the physician follow-up visit in current period, and with OHIP speciality SPEC = 00 |
| Time to homecare visit | HCD, DAD | Service date – discharge date |
| Death within 30 days of discharge | RPBD | Death date |
| Emergency Department visit within 30 days of discharge | NACRS | At least one Emergency Department visit in the thirty days following hospital discharge |
| Emergency Department visit after discharge but before first follow-up physician visit | NACRS | Code as 1 if index date < Emergency Department visit < first follow-up date (from primary outcome) |
| Urgent readmission to hospital within 30 days of discharge | DAD | Include only urgent (ADMCAT=U) admissions |
| Transferred to long-term care facility within 30 days of discharge | CCRS | LTC admission date within 30 days of hospital discharge |
| Physician follow-up | OHIP | A billing code from the OHIP database with a location of home/office/phone/unidentified, and a medical specialty code (00-09, 13, 15-20, 22-24, 26, 31, 33-35, 41, 44, 46-48, 60-64), i.e. excluding non-medical practitioner visits. We excluded visits with location E, I, or L (Emergency, Inpatient, or LTC). |
| Location follow-up | OHIP | Office: Location=O  Virtual: Location=P or FEECODE= K080, K081, K082  Home: Location=H or FEECODE= B960, B961, B962, B963, B964, B986, B987, B988, B990, B992, B994, B996, A900, A901 |

**Table 3. Baseline Characteristics of Patients with and without Functional Decline in the Pre-COVID-19 cohort.**

| **Variable** | **Functional Decline (N=15,637)** | **No Functional Decline (N=6,134)** | **SMD^1^** |
| --- | --- | --- | --- |
| Age (Mean, SD) | 81.53 (8.4) | 79.65 (8.5) | 0.22 |
| Female (n, %) | 9,217 (58.9) | 3,214 (52.4) | 0.13 |
| Rural Status (n, %)   - Large urban - Small urban - Rural - Missing | 10,038 (64.2)  4,164 (26.6)  1,293 (8.3)  142 (0.9) | 3,753 (61.2)  1,708 (27.8)  588 (9.6)  85 (1.4) | 0.06  0.03  0.05  0.05 |
| Income Quintile (n, %)   - 1 (lowest) - 2 - 3 - 4 - 5 (highest) - Missing | 4,140 (26.5)  3,709 (23.7)  2,955 (18.9)  2,506 (16.0)  2,277 (14.6)  50 (0.3) | 1,796 (29.3)  1,393 (22.7)  1,117 (18.2)  951 (15.5)  856 (14.0)  21 (0.3) | 0.06  0.02  0.02  0.01  0.02  0 |
| Arrived by ambulance (n, %) | 10,503 (67.2) | 3,785 (61.7) | 0.11 |
| Charlson Index Group (n, %)   - 0 - 1 - 2 - 3 - 4+ | 5,347 (34.2)  4,269 (27.3)  2,918 (18.7)  1,910 (12.2)  1,193 (7.6) | 2,006 (32.7)  1,678 (27.4)  1,308 (21.3)  740 (12.1)  402 (6.6) | 0.03  0  0.07  0.01  0.04 |
| Dementia (n, %) | 4,338 (27.7) | 1,294 (21.1) | 0.16 |
| CHF (n, %) | 4,213 (26.9) | 1,801 (29.4) | 0.05 |
| COPD (n, %) | 6,092 (39.0) | 2,688 (43.8) | 0.10 |
| Length of stay (Mean, SD) | 12.75 (14.0) | 10.02 (16.0) | 0.18 |
| Acute Length of Stay (Mean, SD) | 10.18 (8.3) | 8.13 (7.0) | 0.27 |
| Discharged on a weekend (n, %) | 5,270 (33.7) | 2,106 (34.3) | 0.01 |
| Discharged with homecare services (n, %) | 14,244 (91.1) | 5,402 (88.1) | 0.10 |
| Homecare usage in 6 months prior to admission (n, %) | 5,842 (37.4) | 2,345 (38.2) | 0.02 |
| Previous Emergency Department visit in 6 months prior to admission (n, %) | 9,521 (60.9) | 3,803 (62.0) | 0.02 |
| Hospital discharge in 6 months prior to admission (n, %) | 1,471 (9.4) | 745 (12.1) | 0.09 |
| Interpreter needed (n, %) | 1,505 (9.6) | 328 (5.3) | 0.16 |
| Primary language not French or English (n, %) | 2,481 (15.9) | 557 (9.1) | 0.21 |
| Living alone (n, %) | 4,865 (31.1) | 2,226 (36.3) | 0.11 |
| Living in a private home or apartment (n, %) | 13,039 (83.4) | 5,338 (87.0) | 0.10 |
| No informal helper (n, %) | 339 (2.2) | 223 (3.6) | 0.09 |
| Supervision or assistance needed with ADLs/IADLS (n, %)   - Bathing - Personal hygiene - Dressing lower body - Locomotion - Meal preparation - Ordinary housework - Managing medications - Stairs | 13,155 (84.1)  6,692 (42.8)  10,666 (68.2)  8,379 (53.6)  13,395 (85.7)  14,714 (94.1)  7,946 (50.8)  13,164 (84.2) | 2,944 (48.0)  1,326 (21.6)  2,009 (32.8)  1,388 (22.6)  3,652 (59.5)  4,463 (72.8)  2,126 (34.7)  3,316 (54.1) | 0.83  0.47  0.76  0.67  0.61  0.60  0.33  0.69 |
| Ability to understand others (n, %)   - Understands - Usually understands - Often/sometimes/rarely/never understands | 9,420 (60.2)  3,875 (24.8)  2,342 (15.0) | 4,556 (74.3)  1,108 (18.1)  470 (7.7) | 0.30  0.16  0.23 |
| Instability of medical conditions (n, %) | 10,977 (70.2) | 3,060 (49.9) | 0.42 |
| One or more falls in last 90 days (n, %) | 8,353 (53.4) | 2,275 (37.1) | 0.33 |
| Top 20 most responsible diagnoses (n, %)   - Congestive heart failure - Urinary tract infection - COPD exacerbation - COPD with acute lower respiratory infection - Pneumonia - Acute renal failure - Delirium - NSTEMI - Femoral neck fracture - Cellulitis - Dementia - Cerebral infarction, unspecified - Cerebral infarction due to occlusion/stenosis of cerebral artery - Delirium superimposed on dementia - Intertrochanteric fracture - Atrial fibrillation - Convalescence following surgery - Malaise and fatigue - Falls - Sepsis | 1,028 (6.6)  512 (3.3)  391 (2.5)  354 (2.3)  414 (2.6)  274 (1.8)  269 (1.7)  244 (1.6)  294 (1.9)  194 (1.2)  246 (1.6)  196 (1.3)  210 (1.3)  212 (1.4)  216 (1.4)  148 (0.9)  137 (0.9)  170 (1.1)  167 (1.1)  152 (1.0) | 412 (6.7)  214 (3.5)  210 (3.4)  222 (3.6)  147 (2.4)  129 (2.1)  93 (1.5)  107 (1.7)  31 (0.5)  127 (2.1)  66 (1.1)  82 (1.3)  52 (0.8)  57 (0.9)  29 (0.5)  89 (1.5)  79 (1.3)  44 (0.7)  47 (0.8)  57 (0.9) | 0.01  0.01  0.05  0.08  0.02  0.03  0.02  0.01  0.13  0.07  0.04  0.01  0.05  0.04  0.10  0.05  0.04  0.04  0.03  0 |

^1^SMD= Standardized Mean Difference

**Table 4. Baseline Characteristics of Patients with and without Functional Decline in the COVID-19 Cohort.**

| **Variable** | **Functional Decline (N=12,965)** | **No Functional Decline (N=4,283)** | **SMD^1^** |
| --- | --- | --- | --- |
| Age (Mean, SD) | 81.18 (8.3) | 79.28 (8.3) | 0.23 |
| Female (n, %) | 7,505 (57.9) | 2,190 (51.1) | 0.14 |
| Rural Status (n, %)   - Large urban - Small urban - Rural - Missing | 8,174 (63.0)  3,499 (27.0)  1,147 (8.8)  145 (1.1) | 2,336 (54.5)  1,357 (31.7)  537 (12.5)  53 (1.2) | 0.17  0.10  0.12  0.01 |
| Income Quintile (n, %)   - 1 (Lowest) - 2 - 3 - 4 - 5 (Highest) - Missing | 3,332 (25.7)  2,955 (22.8)  2,492 (19.2)  2,146 (16.6)  2,004 (15.5)  36 (0.3) | 1,234 (28.8)  936 (21.9)  807 (18.8)  703 (16.4)  593 (13.8)  10 (0.2) | 0.07  0.02  0.01  0  0.05  0.01 |
| Arrived by ambulance (n, %) | 9,330 (72.0) | 2,745 (64.1) | 0.17 |
| Charlson Index Group (n, %)   - 0 - 1 - 2 - 3 - 4+ | 4,541 (35.0)  3,462 (26.7)  2,460 (19.0)  1,571 (12.1)  931 (7.2) | 1,406 (32.8)  1,082 (25.3)  893 (20.8)  548 (12.8)  354 (8.3) | 0.05  0.03  0.05  0.02  0.04 |
| Dementia (n, %) | 2,316 (17.9) | 577 (13.5) | 0.12 |
| CHF (n, %) | 2,674 (20.6) | 989 (23.1) | 0.06 |
| COPD (n, %) | 4,341 (33.5) | 1,678 (39.2) | 0.12 |
| Length of Stay (Mean, SD) | 13.53 (15.4) | 10.22 (20.2) | 0.18 |
| Acute Length of Stay (Mean, SD) | 10.88 (8.9) | 8.49 (7.5) | 0.29 |
| Weekend Discharge (n, %) | 4,379 (33.8) | 1,474 (34.4) | 0.01 |
| Discharged with home services (n, %) | 11,731 (90.5) | 3,757 (87.7) | 0.09 |
| Homecare usage in 6 months prior to admission (n, %) | 4,648 (35.9) | 1,486 (34.7) | 0.02 |
| Previous Emergency Department visit in 6 months prior to admission (n, %) | 7,390 (57.0) | 2,455 (57.3) | 0.01 |
| Hospital discharge in 6 months prior to admission (n, %) | 1,142 (8.8) | 485 (11.3) | 0.08 |
| Interpreter needed (n, %) | 1,208 (9.3) | 228 (5.3) | 0.15 |
| Primary language not French or English (n, %) | 1,988 (15.3) | 408 (9.5) | 0.18 |
| Living alone (n, %) | 3,965 (30.6) | 1,520 (35.5) | 0.10 |
| Living in a private home or apartment (n, %) | 11,032 (85.1) | 3,836 (89.6) | 0.14 |
| Absent informal helper (n, %) | 294 (2.3) | 165 (3.9) | 0.09 |
| Supervision or assistance needed with ADLs/iADLS (n, %)   - Bathing - Personal hygiene - Dressing lower body - Locomotion - Meal preparation - Ordinary housework - Managing medications - Stairs | 11,032 (85.1)  6,012 (46.4)  9,199 (71.0)  7,379 (56.9)  11,327 (87.4)  12,280 (94.7)  6,964 (53.7)  11,154 (86.0) | 2,016 (47.1)  869 (20.3)  1,426 (33.3)  1,013 (23.7)  2,580 (60.2)  3,099 (72.4)  1,508 (35.2)  2,425 (56.6) | 0.88  0.58  0.81  0.72  0.65  0.63  0.38  0.69 |
| Ability to understand others (n, %)   - Understands - Usually understands - Often/sometimes/rarely/never understands | 7,315 (56.4)  3,449 (26.6)  2,201 (17.0) | 3,104 (72.5)  831 (19.4)  348 (8.1) | 0.34  0.17  0.27 |
| Instability of medical conditions (n, %) | 9,553 (73.7) | 2,306 (53.8) | 0.42 |
| One or more falls in last 90 days (n, %) | 7,418 (57.2) | 1,569 (36.6) | 0.42 |
| Top 20 most responsible diagnoses (n, %)   - Congestive heart failure - Urinary tract infection - Delirium - Femoral neck fracture - Acute renal failure - COVID-19 - COPD exacerbation - Intertrochanteric fracture - COPD with acute lower respiratory infection - Pneumonia - NSTEMI - Delirium superimposed on dementia - Cerebral infarction due to occlusion/stenosis of cerebral artery - Cerebral infarction, unspecified - Dementia - Cellulitis - Convalescence following surgery - Atrial fibrillation - Malaise and fatigue - Sepsis | 669 (5.2)  466 (3.6)  287 (2.2)  309 (2.4)  244 (1.9)  274 (2.1)  196 (1.5)  296 (2.3)  186 (1.4)  200 (1.5)  180 (1.4)  215 (1.7)  204 (1.6)  182 (1.4)  190 (1.5)  143 (1.1)  133 (1.0)  129 (1.0)  137 (1.1)  116 (0.9) | 284 (6.6)  173 (4.0)  63 (1.5)  36 (0.8)  98 (2.3)  53 (1.2)  117 (2.7)  16 (0.4)  103 (2.4)  87 (2.0)  74 (1.7)  34 (0.8)  42 (1.0)  63 (1.5)  46 (1.1)  79 (1.8)  50 (1.2)  52 (1.2)  34 (0.8)  50 (1.2) | 0.06  0.02  0.06  0.12  0.03  0.07  0.09  0.17  0.07  0.04  0.03  0.08  0.05  0.01  0.04  0.06  0.01  0.02  0.03  0.03 |

^1^SMD= Standardized Mean Difference
